# Letter to the Editor: AI in radiology: Europe’s prioritization of safety vs Taiwan’s efficiency-driven pragmatism

**DOI:** 10.1186/s13244-026-02440-z

**Published:** 2026-11-11

**Authors:** Wing P. Chan, Russell O. Kosik, Vu Pham Thao Vy, Merel Huisman

**Affiliations:** 1 Department of Radiology, School of Medicine, College of Medicine, Taipei Medical University, Taipei, Taiwan; 2 Department of Radiology, Wan Fang Hospital, Taipei Medical University, Taipei, Taiwan; 3 International Ph.D. Program in Medicine, College of Medicine, Taipei Medical University, Taipei, Taiwan; 4 Department of Radiology, Thai Nguyen National Hospital, Thai Nguyen City, Vietnam; 5 Department of Radiology and Nuclear Medicine, Radboud University Medical Center, Nijmegen, The Netherlands

“We need these rules for AI to move forward”, Emmanuel Macron stated at the AI Action Summit in Paris on February 11, 2025. These words encapsulate the principle of the European Union (EU)’s 2024 Artificial Intelligence (AI) Act, which classifies radiology AI systems as “high risk”. The AI Act establishes a risk-based legal framework to ensure safety, transparency, and respect for fundamental rights while also supporting innovation.

In contrast, Taiwan has prioritized workforce optimization and operational efficiency, favoring post-implementation evaluation over extensive ex ante regulation. These differing milieus have produced distinct innovation pathways. By comparing the EU’s model focusing on “Regulations and Safety” with Taiwan’s “Pragmatic Efficiency”, this article offers a structured policy analysis of the respective strengths and trade-offs. Such debates on innovation and regulation can offer benefits by examining implementation experiences beyond Europe. Taiwan is an instructive comparison, given its comparable demographics and advanced healthcare infrastructure but contrasting regulatory culture.

## Workforce drivers: safety vs pragmatism

A key factor underlying the divergence between safety and pragmatism is healthcare workforce capacity. Although the EU and Taiwan have comparable economic and demographic profiles (mean GDP per capita: USD 32,440 for the EU vs USD 34,162 for Taiwan), their medical workforce dynamics differ substantially. The EU averages approximately 410 physicians and 13 radiologists per 100,000 people [1], compared to Taiwan’s 232 physicians and 5.8 radiologists per 100,000 people, despite near-universal health coverage at 99.9% [2].

In the EU, regulatory frameworks, such as the EU AI Act, are designed to protect core principles, including patient safety, autonomy, and privacy. The EU is comprised of a heterogeneous group of national healthcare systems with varying payer structures, referral pathways, reimbursement policies, and procurement mechanisms, which may contribute to slower and less uniform AI integration across member states. This more fragmented country-level governance structure in the EU contrasts with Taiwan’s relatively centralized healthcare coordination. In Taiwan, AI adoption stems from the practical need to manage increasing workloads. Government-funded programs providing free mammography and low-dose chest CT (LDCT) screening have increased imaging volumes nationwide [3, 4]. In this context, Taiwan has relied on AI tools to support services and clinical efficiency under resource constraints.

## Deployment pathways: integrated screening vs infrastructure building

In Taiwan, AI adoption has been integrated with government-mandated screening programs. Computer-aided detection (CAD) is used in mammography at over 90% of hospitals, primarily to improve reading efficiency. CAD has been used for over two decades to detect asymmetries and microcalcifications. Although its clinical value remains a topic of discussion, approximately two-thirds of radiologists have incorporated CAD as a routine “second check”. Despite modest evidence on CAD, Taiwan’s mammography screening program boasts high recall accuracy (8.15% vs 2.3–13% in the EU) [5]; meaningful reductions in breast cancer mortality, particularly among women aged 45–64; and a 10-year survival rate exceeding 97% [6]. Although these population-level outcomes compare favorably with OECD averages, potential confounders should be considered when making direct comparisons [7–9].

In the EU, AI implementation has followed a different trajectory. AI development has advanced primarily through publicly funded, multi-center initiatives and collaborative infrastructure, including the SOLACE lung cancer screening project [10] and the European Cancer Imaging Initiative [11]. Several European institutions have implemented AI tools to support routine clinical workflows, including automated large-vessel occlusion (LVO) detection in acute stroke, fracture detection on radiographs, and pulmonary nodule detection on chest CT [12]. These examples demonstrate that the EU is actively translating AI into clinical practice despite its stronger regulatory emphasis. EU initiatives such as the “European AI-powered advanced screening centers” program and the broader EU Apply AI strategy for health further support the adoption of clinical AI tools [13]. However, translation into routine clinical practice has progressed more gradually than in Taiwan. This partially reflects the diversity of healthcare systems across EU member states and the rigorous validation and compliance requirements introduced by the AI Act. As a result, the EU has taken a leading role in defining standards for safe and accountable AI. Taiwan, however, offers practical experience in large-scale clinical deployment, with post-deployment evaluation prioritized less or deferred.

## Commercial pathways and reimbursement

Government-supported screening programs in Taiwan have encouraged companies to develop AI solutions for high-volume imaging, particularly chest CT [14, 15]. Unlike mammography, which remains largely qualitative, LDCT lung cancer screening relies heavily on quantitative image assessment. Thus, AI tools for LDCT have been widely adopted, with over 140 hospitals using them and approximately two-thirds of radiologists incorporating AI into pulmonary nodule detection workflows. These tools are associated with gains in efficiency and diagnostic consistency, although challenges remain regarding integration of AI tools into existing PACS-based radiology workflows. Recent implementation-focused reviews highlight medicoeconomic barriers, including limited objective data on return investment, and immature reimbursement pathways [16, 17].

Additional emerging screening applications, including for dementia and osteoporosis [18, 19], further illustrate Taiwan’s distinctive “self-pay” model [20]. In these areas, AI tools are frequently offered as optional out-of-pocket add-ons. This payment structure enables earlier clinical availability but leads to unequal access due to financial barriers for low-income populations. In comparison, adoption in many European settings often depends on hospital budgets or targeted research funding, which can limit immediate access for the general population.

## The price of efficiency: risks and governance

Taiwan’s rapid adoption of AI raises concerns highlighted in EU debates. One concern is algorithmic bias: AI models trained primarily on Taiwanese datasets may have limited generalizability, and models developed on Western populations may perform less reliably on Asian cohorts [21]. Moreover, high screening volumes may increase the risk of automation bias, where busy radiologists rely excessively on AI without sufficient image review [22].

Data governance remains an evolving area. Taiwan’s National Health Insurance database provides a valuable resource for AI development; however, patient consent mechanisms and liability frameworks differ significantly from those in the EU’s General Data Protection Regulation. In anticipation of expanded AI use, Taiwan’s National Science and Technology Council released a Draft AI Basic Law on July 15, 2024. This draft includes 18 articles without defined penalties. Meanwhile, the EU framework comprises 113 articles with enforceable sanctions. Taiwan’s approach reflects a regulatory philosophy that prioritizes adaptability and iterative development, in contrast to the EU’s comprehensive and legally prescriptive framework. At the same time, European guidance increasingly emphasizes continuous post-market surveillance and lifecycle monitoring beyond initial assessment alone [23, 24].

## Conclusion: mutual learning

Taiwan’s integration of AI into government-led screening programs has been associated with sustained accessibility and high participation, while supporting radiology workflow under increasing demand. The EU emphasizes regulatory oversight and formal risk management. Rather than viewing these as opposing models, mutual learning can be garnered around three domains: workflow integration and procurement, lifecycle monitoring and post-market surveillance, and reimbursement/value assessment. Europe may draw on Taiwan’s experience integrating AI as a “second reader” to support radiologists amid workforce constraints. Conversely, Taiwan may benefit from analyzing the EU’s structured frameworks on risk classification to help ensure that efficiency-driven adoption is accompanied by long-term safety and trust. Future governance of radiology AI may require hybrid regulatory models combining centralized oversight with institution-level flexibility, adaptive approval pathways enabling iterative AI model updates under continuous clinical validation, and post-market AI monitoring systems emphasizing real-world auditing, bias surveillance, safety evaluation, and longitudinal performance assessment.

## Data Availability

Not applicable.

## References

[1] Brady AP, Paulo G, Brkljacic B, Loewe C, Szucsich M, Hierath M (2025) Current status of radiologist staffing, education and training in the 27 EU member states. Insights Imaging 16:59. 10.1186/s13244-025-01925-7 PMC11910488 40088348

[2] Organisation for Economic Co-operation and Development (2023) Health at a glance 2023: OECD indicators. OECD Publishing, Paris. 10.1787/7a7afb35-en

[3] Ko CH, Chien LN, Chiu YT, Hsu HH, Wong HF, Chan WP (2024) Demands for medical imaging and workforce Size: a nationwide population-based study, 2000–2020. Eur J Radiol 172:111330. 10.1016/j.ejrad.2024.111330 38290203

[4] Huang C-C, Effendi FF, Kosik RO et al (2023) Utilization of CT and MRI scanning in Taiwan, 2000–2017. Insights Imaging 14:23. 10.1186/s13244-023-01364-2 PMC9897145 36735153

[5] Peintinger F (2019) National breast screening programs across Europe. Breast Care 14:354–358. 10.1159/000503715 PMC6940461 31933580

[6] Giaquinto AN, Sung H, Miller KD et al (2022) Breast cancer statistics, 2022. CA Cancer J Clin 72:524–541. 10.3322/caac.21754 36190501

[7] Yao MM-S, Vy VPT, Chen TH-H et al (2023) Performance measures of 8,169,869 examinations in the National Breast Cancer Screening Program in Taiwan, 2004–2020. BMC Medicine 21:497. 10.1186/s12916-023-03217-7 PMC10724902 38102671

[8] Shen C-T, Hsieh H-M, Tsao Y-H (2024) Socioeconomic inequalities in uptake of outreach mammography before and after accessibility improvement of Taiwan’s national universal breast cancer screening policy. BMC Public Health 24:2439. 10.1186/s12889-024-19896-7 PMC11382483 39245764

[9] Parng I-M (2024) Cancer control HEALTHY TAIWAN 2024. National Health Insurance Administration, Ministry of Health and Welfare. Available via PMDA. https://www.pmda.go.jp/files/000271384.pdf

[10] Kauczor HU, von Stackelberg O, Nischwitz E et al (2024) Strengthening lung cancer screening in Europe: fostering participation, improving outcomes, and addressing health inequalities through collaborative initiatives in the SOLACE consortium. Insights Imaging 15:252. 10.1186/s13244-024-01814-5 PMC11496428 39436577

[11] Martí-Bonmatí L, Blanquer I, Tsiknakis M et al (2025) Empowering cancer research in Europe: the EUCAIM cancer imaging infrastructure. Insights Imaging 16:47. 10.1186/s13244-025-01913-x PMC11850660 39992532

[12] Sarhan K, Azzam AY, Moawad MHED, Serag I, Abbas A, Sarhan AE (2025) Automated emergent large vessel occlusion detection using viz.ai software and its impact on stroke workflow metrics and patient outcomes in stroke centers: a systematic review and meta-analysis. Transl Stroke Res 16:2258–2271. 10.1007/s12975-025-01354-0 PMC12596299 40335883

[13] European Commission (2025) Apply AI strategy. Available via EU. https://digital-strategy.ec.europa.eu/en/policies/apply-ai. Accessed 19 May 2026

[14] Lin TM, Yang HW, Han CC, Lin CS (2025) Taiwan’s smart healthcare value chain: AI innovation from R&D to industry deployment. Healthcare (Basel). 10.3390/healthcare14010023 PMC12786189 41516953

[15] Taiwan Ministry of Health and Welfare (2024) Taiwan health and welfare report. https://www.mohw.gov.tw/cp-137-79807-2.html

[16] Organisation for Economic Co-operation and Development (2026) Progress in implementing the European Union coordinated plan on artificial intelligence, Vol. 2. In: Uptake in high-impact sectors. OECD Publishing, Paris. 10.1787/3ac96d41-en

[17] Bamberg F, Adam G, Antoch G et al (2026) Artificial intelligence in radiology: unlocking new dimensions of value. Rofo. 10.1055/a-2794-9496 41839210

[18] Hsieh WT, Groot TM, Yen HK et al (2024) Validation of ten osteoporosis screening tools in rural communities of Taiwan. Calcif Tissue Int 115:507–515. 10.1007/s00223-024-01273-6 39155291

[19] Lyu W-J, Chiu P-Y, Liu C-H, Liao Y-C, Chang H-T (2024) Determining optimal cutoff scores of Cognitive Abilities Screening Instrument to identify dementia and mild cognitive impairment in Taiwan. BMC Geriatrics 24:216. 10.1186/s12877-024-04810-y PMC10909252 38431549

[20] Shih DP, Kuo HW, Liang WM, Lin PY, Tseng P, Wang JY (2021) Association of health checkups with health-related quality of life among public servants: a nationwide survey in Taiwan. Health Qual Life Outcomes 19:42. 10.1186/s12955-021-01684-1 PMC7863473 33541360

[21] Colacci M, Huang YQ, Postill G et al (2025) Sociodemographic bias in clinical machine learning models: a scoping review of algorithmic bias instances and mechanisms. J Clin Epidemiol. 10.1016/j.jclinepi.2024.111606 39532254

[22] Kim SH, Schramm S, Riedel EO et al (2025) Automation bias in AI-assisted detection of cerebral aneurysms on time-of-flight MR angiography. Radiol Med 130:555–566. 10.1007/s11547-025-01964-6 PMC12008054 39939458

[23] Cardoso MJ, Moosbauer J, Cook TS et al (2023) RAISE—radiology AI safety, an end-to-end lifecycle approach. Preprint at https://arxiv.org/abs/2311.14570

[24] Cuocolo R, Bernardini D, Pinto dos Santos D et al (2025) AI medical device post-market surveillance regulations: consensus recommendations by the European Society of Radiology. Insights Imaging 16:275. 10.1186/s13244-025-02146-8 PMC12701188 41385025

